# Forestalling the Epidemics of Parkinson's Disease Through Plant-Based Remedies

**DOI:** 10.3389/fnut.2018.00095

**Published:** 2018-10-30

**Authors:** Ines Banjari, Tihana Marček, Svetlana Tomić, Viduranga Y. Waisundara

**Affiliations:** ^1^Department of Food and Nutrition Research, Faculty of Food Technology Osijek, Josip Juraj Strossmayer University of Osijek, Osijek, Croatia; ^2^Department of Neurology, Osijek University Hospital Center, Osijek, Croatia; ^3^Department of Food Technology, Faculty of Technology, Rajarata University of Sri Lanka, Mihintale, Sri Lanka

**Keywords:** Asia, Europe, complementary and alternative therapies, parkinson's disease, traditional medicinal herbs

## Abstract

Parkinson's disease (PD) as the second leading neurodegenerative disease, imposes a heavy burden among individuals as well as economies worldwide. The main characteristics of PD is a progressive loss of dopaminergic neurons resulting in the loss of motor function, the occurrence of non-motor symptoms, and cognitive decline. Similar to many other chronic diseases, complementary and alternative therapies (CAT) are very popular for the treatment of this disease. This review evaluates six plants, three each from European and Asian traditional medicinal systems: (1) *Atropa belladonna*, (2) *Hyoscyamus niger*, (3) *Lepidium meyenii*, (4) *Aspargus racemosus*, (5) *Mucuna pruriens* L., and (6) *Gingko biloba*. *Atropa belladonna*, and *Hyoscyamus niger* in particular, are better known for their poisonous and narcotic effects than as potentially effective plants for the treatment of neurodegenerative diseases. *Ginkgo biloba* is one of the most widely cultured plants in Traditional Chinese Medicine with high antioxidant potential which contributes to its neuroprotective/ anti-apoptotic activity. The bioactive compounds, anti-neurodegenerative effects and other neuroprotective effects of all six plants are discussed herein.

## Introduction

Parkinson's disease (PD) is the second most common neurodegenerative disease globally. Currently, approximately 6 million people worldwide or around 1% among those over 60 years of age are affected by PD (1). Recent meta-analysis revealed a rising prevalence of PD with age across regions (2), where the prevalence is predicted to double by the year 2030 (3). The economic burden of countries based on those contracted with PD will continue to rise as well. Estimated direct cost of PD in Europe in 2005 was €10.7 billion per year (4), while current estimates for the United States mention that combined direct and indirect costs of PD are $25 billion per year (5). The economic cost increases as PD progresses (6, 7), and the medical care costs are the principal cost burden of PD patients (6, 7). On a comparative basis, the prevalence is significantly higher in North America, Europe, and Australia than in Asia, and males seem to be more susceptible than females (2).

The main characteristic of PD is a progressive loss of dopaminergic neurons resulting in the loss of motor function, non-motor symptoms, and cognitive decline (1, 3, 8). The 3 major symptoms of PD are tremor, rigidity and bradykinesia (1, 5, 8). Changes in taste and smell perception, clinical depression, gastro-intestinal dysfunction and sleep disturbance are the most important non-motor symptoms (5), and for some, early symptoms of PD appear long before the onset of the disease (9). The exact etiology of the disease is still unknown (1, 10, 11). Around 10% of PD cases are found to be triggered by mutations in the alpha-synuclein, leucine-rich repeat kinase 2, Parkin, PINK, LRRK2 and several other genes (10, 11). As for the remaining 90% of PD cases which are sporadic (8), the combined effect of environmental exposure and genetic susceptibilities are believed to play a central role in the disease progression (8, 11). Along with aging, exposure to various environmental pollutants (especially pesticides), lack of sleep, obesity and a diet low in antioxidants are most extensively discussed as associated causes of PD (8, 12).

Similar to many other chronic diseases for which no cure exists and with high economic burden on both the individuals and the society, complementary and alternative therapies (CAT) are a very popular method of combating PD. Some of the CAT include physical activity (especially Yoga and Tai chi), traditional herbs, dietary supplements, acupuncture and molecular targeted therapies (13, 14). Between 25.7 and 76% PD patients reach out to CAT with the aim of improving the associated motor symptoms. However, it is interesting to note that CAT is even recommended by health professionals up to 20% of times during patient consultations (15, 16).

The aim of this short review is to summarize the existing evidence on some selected plants used in traditional medicine for the treatment of PD. The paper focuses on plants from Europe and Asia which have been used for treating neurodegenerative diseases as a whole, as well as been extensively and holistically used around the globe, both as medical remedies or functional foods (i.e. bioactive components in various functional foods). Although many recent reviews elaborate the antioxidant potential of various plant-derived bioactive compounds for the treatment of PD, the pathology of the disease itself is complex and not fully elucidated, and it is doubtful whether the antioxidant potential of plants would be the sole mechanism of action which would combat the disease progression. Thus, in view of this lack of evidence to put the sole responsibility on oxidative stress as the root cause of PD, we explored other potential anti-parkinsonian mechanisms of the selected plants in this review and their bioactive compounds responsible for these pathways.

## Search strategy and selection criteria

The focus of our paper were plants used for the treatment of PD, and only plants tested in humans. As noted previously, plants with mechanisms of action beyond simply demonstrating antioxidant activities were selected.

The methodology for systematic reviews was modified and divided into a two stage process owing to the availability of references and the languages in which they are written. For the European plants, due to the lack of systematic databases on herbs traditionally used to treat PD, the first stage focused on hard-cover books published in Croatia and surrounding countries (ex-Yugoslavia) to prepare the list of herbs used in Europe, and the oldest book used was Gursky (17). For herbs used in Asia, the same strategy was used, although the unavailability of books published in English was a challenge. Most of the books which were referred for this review were published in either Sinhala or Tamil, which are two native languages of Sri Lanka. Nevertheless, in order to avoid translational errors and misinterpretations, occasional referencing of Sinhala and Tamil books for verification purposes was carried out while primary sourcing was done in books published in English.

The list of plants retrieved from literature was then selected on the basis of the highest number of citations, which were then used for the second stage of the review. A search was conducted in the following databases: HRCAK (Croatian database), Pub Med, Cochrane, Medline Plus, ScienceDirect, Web of Science and Google Scholar. The following key words were used as the search terms: Ayurveda, *Asparagus racemosus, Atropa belladonna, Gingko biloba, Hyoscyamus niger, Lepidium meyenii, Mucuna pruriens*, Europe, Asia, herbs, herbal remedies, traditional medicine, Parkinson disease, neurodegenerative disease. The last search was conducted on July 18, 2018. Additionally, we searched the largest database of clinical trials (ClinicalTrials.gov) to identify whether any randomized clinical trial fits within the criteria. The last search was conducted on June 29, 2018 but out of three trials, none fit the criteria. Table 1 provides a summary of the plants, the part of the plant used for the treatment, their bioactive compounds, anti-neurodegenerative effects and other neuroprotective effects.

**Table 1:** Bioactive compounds with neuroprotective effects isolated from European and Asian plants.

**Plant**	**Plant part**	**Bioactive compound**	**Anti-neurodegenerative effects**	**Other neuroprotective effects**
**European**				
*Atropa belladona*	Root	Nicotine		Cholinergic, dopaminigenic, serotoninergic
		Atropine	Antiparkinsonian	Vasodilatator, CNS depressant and stimulant, anticholinergic
		Choline	Antialzheimerman (6–16 g/person/day), antidementia	Cholinergic, hypotensive, memorigenic, cerebrotonic
		Homatropine		Anticholinergic, antiganglionic
		Hyoscyamine	Antiparkinsonian	CNS depressant and stimulant, anticholinergic, antineuralgic
		Phytosterols		Hypocholesterolemic
		Pyridine		CNS depressant
		Scopolamine	Antiparkinsonian	CNS depressant, anticholinergic, anticonvulsant, antiinflammatory
		Scopoletin		CNS depressant and stimulant, antioxidant, anticholinergic, hypotensive, anti-inflammatory
		Tannin		Antioxidant
		Umbelliferone		Anti-inflammatory
*Hyoscyamus niger*	Plant	Choline	Antialzheimeran 5-16 g/person/day, antidementia, memorigenic	Hypotensive, cerebrotonic, cholinergic
		Coumarin		Anti-inflammatory
		Esculetin		Anti-inflammatory
	Leaf	Atropine	Antiparkinsonian	Anticholinergic, CNS depressant and stimulant
		Chlorogenic-acid		Antioxidant, anti-inflammatory
		Gaba		Hypotensive 1,000–4,000 mg/day, CNS inhibitor, anticonvulsant
		Hyoscyamine	Antiparkinsonian	Anticholinergic 150–300 ug 4x/day/person, antineuralgic, CNS depressant and stimulant
		Pyridine		CNS depressant
		Rutin	Antidementia	Hypotensive, anticonvulsant, antioxidant, anti-inflammatory
		Trimethylamine		Antioxidant
	Seed	Atropine	Antiparkinsonian	Anticholinergic, CNS depressant and stimulant
		Linoleic-acid		Anti-inflammatory
		Minerals (calcium, copper, iron, magnesium, manganese, potassium, sodium, zinc)	Antialzheimeran (Zn) 50 mg/day, antidementia (Zn)	Hypotensive (Mg) 260–500 mg/day, (K, Zn 30 mg/day, Ca 1 g/day), Mg antidepressant and CNS depressant, K antidepressant, Ca antidepressant
		Fatty acids (myristic, oleic, palmitic, stearic)		Antioxidant, anti-inflammatory (oleic)
		Scopolamine	Antiparkinsonian	Anticholinergic, CNS depressant, anticonvulsant, anti-inflammatory
*Lepidium meyenii*	Root	Aminoacids (alanine, arginine, glutamic acid, glycine, histidine, leucine, lysine, methionine, phenylalanine, serine, threonine, tryptophan, tyrosine, valine)	Antiparkinsonian 1–5 g/day Methionine, 200–500 mg/day/man Phenylalanine, 2 g 3 x/day Tryptophan, 100 mg/kg/day Tyrosine, antidementia 3 g Tryptophan/day	Antihipertensive (Arginine), Vasodilatator (Arginine), Hypotensive (3 g Tryptophan/day), Antidepressant (50–4,000 mg Phenylalanine/day/person; 1–3 g Tryptophan 3x day/person orl, Tyrosine), Serotoninergic 6–12 g Tryptophan/day/orl/person
		Beta-sitosterol		Antihyperlipoproteinaemic, anti-inflammatory, hypocholesterolemic (2–6 g/person/day orl)
		Campesterol		Hypocholesterolemic
		Phytosterols		Hypocholesterolemic, antidepressant, hypotensive, vasodilatator
		Stigmasterol		Anti-inflammatory, antioxidant, hypocholesterolemic
		Tannin		Antihypertensive
		Vitamins (ascorbic acid, niacin, thiamin, riboflavin, B12)	Antiparkinsonian (1 g 2–3x/day vitamin C, 100 mg/day niacin), antialzheimerman (niacin, 2,000-6,000 mg vit C/day, 100–3,000 mg/day thiamin), antidementia (vitamin C, niacin, thiamin)	Anticonvulsant (niacin 3 g/day), vasodilatator (vitamin C, niacin), hypotensive (vit C 1,000 mg/person/day), hypocholesterolemic (vit C 300–1,000 mg/day; 50–100 mg niacin 3x/day), antihypertensive (vitamin C), anti-inflammatory (vitamin C), antineuralgic (niacin, 1-4 g thiamin/day), antidepressant 2,000 mg vit C/day; serotoninergic (niacin)
**Asian**				
*Asparagus racemosus*	Root	Steroidal saponins	Antiparkinsonioan	
		Isoflavones		
		Asparagamine		Anti-Alzheimers
		Racemosol		
		Isoflavones		
		Saponins		Immunostimulatory
		Immunoside		
*Mucuna pruriens*	Seed	L-3,4-dihydroxy phenylalanine (L-DOPA)		Anti-apoptotic
*Gingko biloba*	Seed			

## European plants

**Figure 1:**
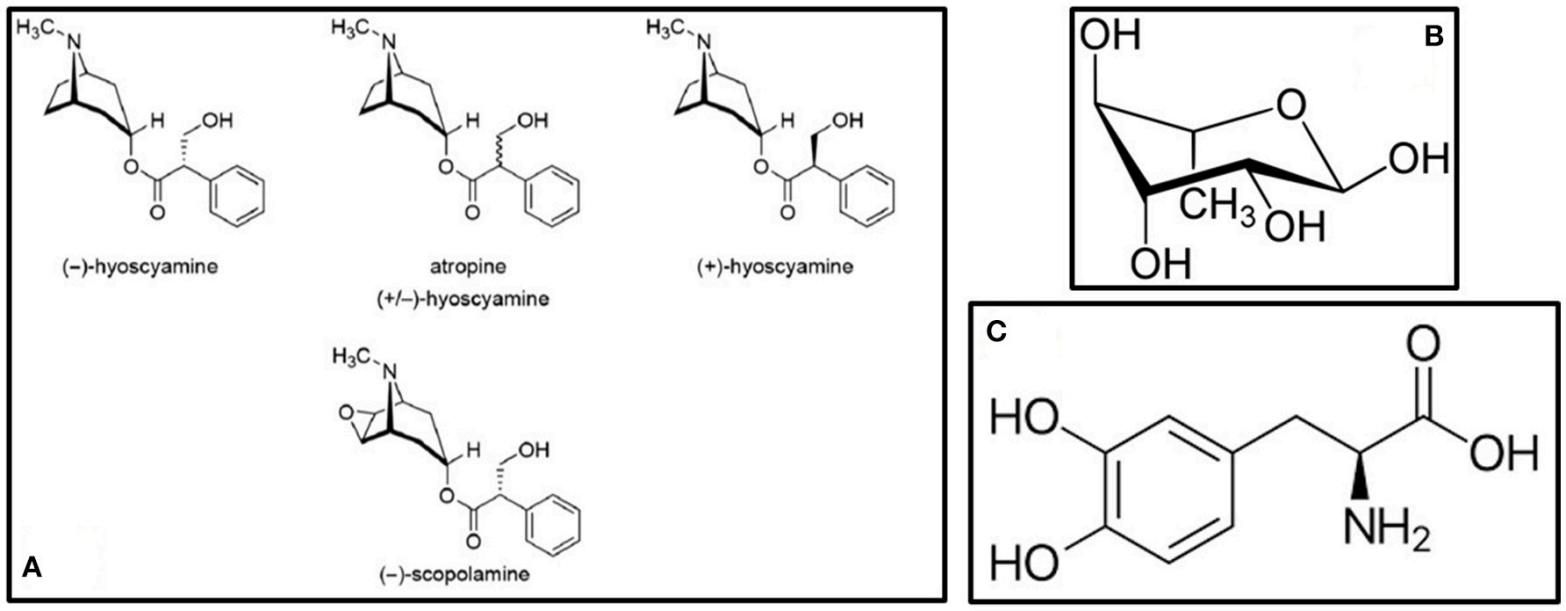
Bioactive compounds present in **(A)** *A. belladona* and *H. niger* (18) **(B)** *A. racemosus* – Rhamnose (19), and **(C)** *Mucuna pruriens* – L-DOPA (20).

*Atropa belladona* and *Hyoscyamus niger* are better known for their poisonous and narcotic effects than as treatments for neurodegenerative diseases. One murder case using *H. niger* in 1910 is even considered as ground-breaking in the science of forensic medicine in the UK (16). In Central European countries however, *A. belladona* and *H. niger* are listed as plants which can be used to treat PD and other forms of neurodegenerative diseases (i.e., Alzheimer's disease and dementia), but with extreme caution and under professional supervision (17). *A. belladona* and *H. niger* share bioactive components that show beneficial effects on PD, some of which are atropine, hyocyamine and scopolamine (18). These are even used as active components in some drugs for PD available on the market (18). The chemical structures of these compounds are shown in Figure 1A. Traditionally, powder prepared from *A. belladona* without the root, and tincture prepared from *H. niger* leaves are used to alleviate the motor symptoms of PD (17). Besides the direct anti-parkinsonian, anti-Alzheimer and anti-dementia effects, both plants contain bioactive components with other neuroprotective effects (Table 1). However, the lack of clinical evidence in the area of PD and other neurodegenerative diseases is substantial.

### *Atropa belladonna* L. (solanaceae; common name: nightshade)

Europe, Asia and North Africa are considered the natural origins of *A. belladona*, with the exceptions of England and Scotland (21, 22). It prefers limestone or chalk grounds, hidden from direct exposure to sunlight (23). The stem is erect, purplish colored, and the root is over 15 cm long, thick and well developed (24). The flower has a campanulate-shaped corolla with five purplish to yellowish-purple painted hanging petals which become lighter in the downward direction. The maturity is reached in the second year with blooms from June maturing in early September, producing the black shiny berry fruits. All parts of the plant, including the kidney-shaped seeds are toxic (23).

Traditionally, *A. belladona* has been used in many cultures around the world as narcotic, anodyne (painkiller), antispasmodic, anticholinergic and diuretic effects, as well as for the treatment of rheumatism, fever, night sweeting, epilepsy, various cancers and mydriasis (dilated pupils) (18). Although poisonous, *A. belladonna* can act as an antidote in case of intoxication with morphine, pilocarpine and muscarine (17). Current medicine is well familiar with *A. belladonna*-based drugs. Atropine, scopolamine, anisodine and anisodamine inhibit the stimulant actions of acetylcholine (ACh) on three levels. In the central nervous system (CNS), they inhibit the muscarinic cholinergic receptors, and inhibit ACh in peripheral structures innervated by the cholinergic nerves and smooth muscles responding to ACh, but without cholinergic innervation (25–27). In large doses, these drugs cause learning and memory deficits, with scopalamine being the most potent (26).

### *Hyoscyamus niger* L. (solanaceae; common name: *black henbane, henbane)*

Natural habitats of *H. niger* are Scandinavia and the northern part of Europe from where it has spread out worldwide such as to North America, Asia and North Africa (28, 29). It is considered as a highly adaptable and invasive species due to high tolerability of temperature and humidity ranges (30). *H. niger* is a vascular plant with great biodiversity and intense fragrance (31). The stem is 30–100 cm, long, unbranched or rarely branched, covered with sticky hairs. It flowers between June and September, and flowers are of pale yellow color with visible purple veins. The fruit is a capsule that opens after maturity, immediately spreading black seeds following ripening (29). All plant parts are poisonous.

Every traditional medicine lists *H. niger* as a medicinal plant. Yet, the most common application is for pain treatment and as a narcotic, nevertheless, with a high frequency of intoxication especially among children (32). *H. niger* has anticolinergic, antisecretory and bronchodilating, spasmolytic, urinary bladder relaxant, hypnotic, hallucinogenic, pupil dilating, sedative, anti-diarrheal, anti-inflammatory, hypotensive, antimicrobial, anticolvunsant, and anticancerogenic effects (18, 33). It has been used to treat PD in Ayurveda as well, although as a minor component of herbal formulations (33). Similar to *A. belladonna*, atropine from *H. niger* is an effective antimuscarinic agent used for the treatment of intoxication with organophosphate compounds (33). In terms of PD treatment, potential mechanisms include lower monoamine oxidase (MAO) activity and protection of neuronal mitochondria from intestinal hydroxyl radical accumulation (34).

### *lepidium meyenii* (brassicaceae; common name: maca, peruvian ginseng)

The origin of *L. meyenii* is South America, primarily Peru highlands, at altitudes from 4,000 to 4,500 m above sea level (34). The tuberous root of *L. meyenii* is the edible part of the plant, with colors ranging from yellow, to red or extremely black (35). It can successfully tolerate wide temperature ranges from −20° to 20°C, high exposure to sunlight and strong winds (36). Depending on the water content and temperatures, *L. meyenii* has the properties of an annual or biennial species (32).

In *L. meyenii*, the neuropotent compounds (Table 1) include amino acids and vitamins which are highly abundant in the root (18). While *L. meyenii* is mainly used as a supplement, native Peruvian population in the central Andes use the hypocotyls as food in an approximate amount of >20 g/d (37). Different varieties of *L. meyenii*, which can be recognized by the color of the hypocotyls, have different biological properties (36, 38). Traditionally, dried *L. meyenii* is commonly boiled or extracted in alcohol before consumption, and the method of preparation influences the content and composition of bioactive compounds (36, 38).

The most common traditional applications of *L. meyenii* as confirmed by clinical trials, are for sexual dysfunction, increased sperm count and motility (36, 39, 40), as well as for the treatment of osteoporosis. *L. meyenii* reduces the number of fractures, regulates glucose and lipid metabolism while consequently reducing blood pressure, and protects the skin against ultraviolet radiation. The red variety of *L. meyenii* has been shown to reverse benign prostatic hyperplasia (35). Though traditionally, *L. meyenii* is not listed as a remedy for learning and memory enhancement, native Peruvians recommend its use in children to improve cognition but without specifying which variety of *L. meyenii* should be used (36). Black *L. meyenii* nevertheless, continuously shows the best results for memory and learning (35, 36, 39). Antioxidant and acetylcholinesterase (AChE) inhibiting activities are the probable reason for black *L. meyenii*'s potential in improving memory (41). On the other hand, antidepressant activity has been found in three varieties of *L. meyenii*: yellow, red and black (42). These activities were confirmed in animal models. The same team of researchers (41) then administered two concentrations of aqueous (0.50 and 2.00 g/kg) and hydroalcoholic (0.25 and 1.00 g/kg) extracts of black *L. meyenii* for 35 days to male mice with scopolamine-induced memory impairment. Both extracts significantly alleviated the scopolamine-induced memory impairment through inhibition of brain AChE activity, but without any effect on MAO activity (41). Recently, *L. meyenii* extract was also found to inhibit butyrylcholinesterase (BuChE) activity (43). Interestingly, the polyphenolic composition of the hydroalcoholic extract of black *L. meyenii* was mentioned as the probable reason for significant improvement of memory impairment induced by ethanol in mice, with a dose–response effect (42). In humans, *L. meyenii* reduces symptoms of depression and anxiety in males, but also reduces psychological symptoms including anxiety and depression in postmenopausal women during a six-week application in amounts of 3.5 g/day (35).

## Asian plants

The Asian plants selected for the review are collectively used in traditional Ayurvedic medicinal systems, primarily in the Indian sub-continent. While many similarities in the bioactive compounds were present in the European herbs, the Asian herbs have not shown such significant overlapping chemical compositions, although L-3,4-dihydroxy phenylalanine (L-DOPA) has been found in both *M. pruriens* and *G. biloba*. This demonstrates the diversity of compounds which can be used for anti-PD effects and the potential to eventually develop medical remedies using herbal combinations for better overall therapeutic outcomes in preventing the disease progression.

### *Aspargus racemosus* (asparagaceae)

Known as Satawar, Satamuli, Satavari, of *A. racemosus* is a woody, perennial climber, a spinous under-shrub reaching a height above 1–2 m. The name Shatavari is an Indian word, meaning “women who have 100 husbands” suggesting its effects on fertility and viability. It is indigenous to India, Sri Lanka and the Himalayas where it can be found at altitudes of 1,300–1,400 m, while the plant is found in Australia, other parts of Asia and Africa in low-shaded altitudes (44). The plant grows well on different soil types from light, sandy to heavy (clay) or rocky, under hot climates with minimum rainfall (45, 46). The upper part of the plant has needle-like leaves, while the flowers are white and scented, and the fruit is a red-colored berry with one seed (47, 48). The root is tuberous, with numerous tubers of lengths of 30–100 cm. The plant blooms during February-March and ripens in April (49, 50).

Almost all parts of *A. racemosus* are used in many of the South Asian traditional medicinal systems for the treatment of various ailments in human beings (51). Classical treatment of using *A. racemosus* include powdering the root (Table 1) and taking in 12 g dosages with 100–250 mL milk twice a day (49). It belongs to the “rasayana” class of herbs which enhance physical and mental health, improve the immune response, and enhance longevity (50). *A. racemosus* showed high free radical scavenging as well as neurotropic modulatory properties in diseases associated with neuron cell loss (51, 52). Administration of the methanolic root extract of *A. racemosus* prevented scopolamine- and sodium nitrite-induced amnesia showing a great potential in memory deficits (53). The anti-PD compounds present in *A. racemosus* include racemosol and rhamnose (19). The chemical structure of rhamnose is shown in Figure 1B. In the study by Jayashree et al. (54), LC–ESI–MS/MS analysis of the methanolic extract of the root of this plant showed the presence of flavonoids, saponins and shatavarins. In this study, the effect of *A. racemosus* extract was assessed in the prevention of tert-Butyl hydroperoxide (t-BHP)-induced damage in Wistar rats. The rats were treated with 1 mmol/kg body weight of t-BHP to induce oxidative stress. Supplementation with *A. racemosus* decreased the level of lipid peroxidation products after t-BHP treatment. *A. racemosus* is used to treat rigidity of limbs and is a memory stimulant (55). The root extract has been observed to contain immunoside, which is a sarsasapogenin glycoside used for immunostimulatory activities (56). Bhatnagar et al. (57) investigated the effect of the aqueous root extract of *A. racemosus* in primary hippocampal neuron cell culture. The extract was shown to be potent when combined with *Withania somnifera* to induce neuroprotection in this particular study (58).

### *Mucuna pruriens* L. (fabaceae)

Also known as velvet bean, cowitch, Bengal velvet bean, cowage, lacuna bean and Lyon bean (English common names), *M. pruriens* is an annual, self-pollinated legume originating from South China, Malaysia and eastern India from where it has spread worldwide and is cultivated as a green vegetable crop (59, 60). According to Padmesh et al. (60), this species has at least three varieties in India; *M. pruriens* (L.) DC, *M. pruriens* (L.) DC var. *pruriens* and *M. pruriens* var. *utilis* (Wall *ex* Wight) Baker *ex* Burck. This is an invasive species, and like other Legume species, it is also known as a weed controller (61). The stem is thin and long and a climber with aggregated flowers which are white in color. The fructus pod has long silky hairs containing 4–6 seeds and the chemical mucunain that causes itchy dermatitis (62, 63). For the full development, the plant requires wet, warm conditions, and flowers between August and April while the pod matures between October and January (30).

Many researchers have found that *M. pruriens* contains L-DOPA, which provides long-term amelioration of PD (63–65). *M. pruriens* had also shown positive effects on PD patients in clinical trials, with a quick onset of action and without concomitant increase in dyskinesia (63, 66). Additionally, Zandopa (HP-200)—a commercial preparation of *M. pruriens* is also available for the treatment of PD (63, 67). In a study by Yadav et al. (68), the neuroprotective effects of an ethanolic extract of *M. pruriens* seeds (Table 1) were evaluated in a Parkinsonian mice model where symptoms were induced by the pesticide paraquat. It was observed that the extract improved the motor abnormalities observed in PD mice, as well as effectively rescued the levels of dopamine and its metabolites in substantia nigra. Furthermore, Yadav et al. (69) evaluated the therapeutic effects of the aqueous extract of *M. pruriens* seed in PD mouse model developed by chronic exposure to paraquat. In this study, it was revealed that the nigrostriatal portion of PD mouse brain showed significantly increased levels of nitrite, malondialdehyde (MDA) and reduced levels of catalase compared with controls. In the study by Poddighe et al. (20), a PTEN-induced putative kinase 1 mutant of *Drosophila melanogaster* were treated with the methanolic extract of *M. pruriens*. Their results showed multiple centers of action, indicating that its effectiveness goes well beyond its L-DOPA content (70). The chemical structure of L-DOPA is shown in Figure 1C. Also, the extract was found to be able to delay the onset of chronic L-DOPA-induced long-term motor complications. As additional treatments extending from this plant, *M. pruriens* can relieve inflammation, neuropathy, nephropathy, delirium, cephalagia, general debility, dysmenorrhea, amenorrhoea, ulcers, constipation, elephantiasis, consumption, helminthiasis, fever and dropsy (70).

### *Ginkgo biloba* (*ginkgoaceae*)

*Ginkgo biloba* is a long-living tree, which can live up to thousand years or more reaching over 30 m in height. The name Ginkgo comes in several name variations such Yajiao (meaning duck foot), Bajguo (white nut), Gongsunshu (grandfather/grandson tree), Icho or Yinxing (silver apricot) or maidenhair tree (71). Eastern China, Zhejiang region, are natural habitats (72). Ginkgo has over 200 cultivars that differ from native species in size, shape and color of the leaves (72).

*Ginkgo biloba* has a special place in Traditional Chinese Medicine as one of the most widely cultured plants; it has antioxidant and free radical scavenger properties (73, 74) which are associated with its neuroprotective/ anti-apoptotic activity (75) (Table 1). Biological activities of the standardized extract of *G. biloba* (EGb761®) have been researched for more than 20 years (76). Nevertheless, given the vast amount of research which is currently available and the extensive usage of this herb in neuroprotective effects, this portion of the review focused only on a few studies which have stood out as updates on the therapeutic properties of the herb.

Recently, Gu et al. (77) evaluated the *G. biloba*'s therapeutic role toward ischemia/ reperfusion (I/R) injury through determination of monoamine neurotransmitter dopamine in corpus striatum in male Sprague-Drawley rats. The mechanism of action is through prevention of excessive release of dopamine in striatum. Wang and Wang (78) observed that it attenuates oxidative stress and apoptosis in mouse cochlear neural stem cells. The extract of this herb is known to contain flavonoids which slow down the oxygen consumption of the stimulated cells by its inhibitory action on NADPH oxidase (79, 80). Amentoflavone has been observed as one of the primary bioactive compounds present in *G. biloba* against PD (81). This compound has shown activity at the allosteric benzodiazepine site of the γ-aminobutyric acid–A receptor (GABA_A_) as a negative allosteric modulator. Polyphenols from *G. biloba*, consisting of namely quercetin and kaemferol, have been shown to have antidepressant-like effects in mice and increased neuronal survival and plasticity through allosteric modulation as well (82, 83). According to Hang et al. (84), EGb761® prevents the formation of apoptosome and the apoptotic cascade by blocking cytochrome-c release.

## Conclusions and future directions

With the growing elderly population around the globe, it is not surprising that healthy aging has become one of the top priorities, with PD at the center of attention. There are many commercial drugs available in the market. However, their inadequacy has led to the search for novel treatments for PD. In view of this flaw, this review highlights the importance of an urgent need to initiate remedial action toward neurological disorders such as PD, at least through CAT. A recent systematic review of randomized controlled trials by Kim et al. (85) did not adequately summarize the evidence on the effectiveness of herbal medicines for PD due to the large heterogeneity between herbal mixtures being used for this disorder as well as the nature of the study designs. Still, the interest in PD is growing, and current experimental evidence does suggest that a number of plants show some neuroprotective potential, well beyond simply demonstrating antioxidant activity. Along with the bioactive compounds described herein, those described by Fu et al. (86), as well as components isolated from *Curcuma longa* (87) and *Panax ginseng* (88, 89) will continue to be in the focus of interest for the development of alternative PD treatments.

Out of plants described here, *A. racemosus* is the most interesting of all because of its great potential to be developed into a herbal remedy and also because it is consumed as a functional food. On the other hand, herbs such as *G. biloba* (EGb761®) have already been commercialized as a potential remedy for neurodegenerative diseases. As highlighted in this review as well as by Campos et al. (90), along with potent neuroprotective effects, (91) many plant bioactive compounds offer diversity which is needed for the holistic recovery from neurodegenerative diseases. More systematic studies are required to elucidate the effectiveness of these plants, including isolation of novel compounds with therapeutic potential. Through such efforts, the burden of the disease upon patients and economies alike could be reduced for the purpose of health and well-being.

## Author contributions

IB, TM, ST, and VW equally contributed to the acquisition of information, drafting the manuscript and approving the final version.

### Conflict of interest statement

The authors declare that the research was conducted in the absence of any commercial or financial relationships that could be construed as a potential conflict of interest.
